# Minimal Criteria to Screen for Wilson Disease: A Delphi Consensus in the United States

**DOI:** 10.1155/ijh/5525442

**Published:** 2025-11-01

**Authors:** Jeff M. Bronstein, Regino P. Gonzalez-Peralta, Matthew Lorincz, Valentina Medici, Sergio Diaz-Mendoza, Krystallia Pantiri

**Affiliations:** ^1^Department of Neurology, David Geffen School of Medicine, University of California, Los Angeles, Los Angeles, California, USA; ^2^Advanced Pediatric Hepatology and Liver Transplantation, AdventHealth for Children, AdventHealth Transplant Institute, Orlando, Florida, USA; ^3^Department of Neurology, University of Michigan, Ann Arbor, Michigan, USA; ^4^Department of Internal Medicine, Division of Gastroenterology and Hepatology, University of California Davis, Sacramento, California, USA; ^5^OPEN Health Group, Marlow, UK; ^6^Illumina, Cambridge, UK; ^7^OPEN Health Group, Rotterdam, Netherlands

## Abstract

**Background:**

The objective was to develop consensus on minimal screening criteria for Wilson disease (WD) diagnosis in US gastroenterology and neurology settings for implementation in clinical practice to support the timely diagnosis of WD.

**Methods:**

A modified Delphi panel with three rounds was conducted. The first round survey was developed with input from a steering committee of four clinical experts in WD who set the analysis rules (consensus: ≥ 80%). Other US gastroenterologists/hepatologists or neurologists with experience treating WD were recruited using purposive sampling, and 32 were invited to participate.

**Results:**

Eleven panelists completed the three rounds. Consensus was reached for 94/126 (74.6%) statements. All panelists agreed that hepatomegaly, splenomegaly, or stigmata of liver disease are suggestive of WD in patients with a neuropsychiatric manifestation; a neurologic exam, 24-h urine copper, ceruloplasmin, and Kayser–Fleischer (KF) ring examination should be performed; and liver biopsy and liver copper determination can be a useful final stage to confirm WD diagnosis. Panelists agreed that noninvasive testing should be performed prior to invasive testing and that the absence of KF rings does not exclude a diagnosis of WD. Panelists agreed that it is important to collaborate in a multidisciplinary team.

**Conclusions:**

This study identified minimal criteria to raise suspicion of WD, minimal tests to confirm or rule out a WD diagnosis, and areas with poor consensus to be explored in future research. These results can complement clinical practice guidance and support cross-specialty collaboration.

## 1. Introduction

Wilson disease (WD) is a rare autosomal recessive condition arising from variants in the ATPase copper transporting beta (*ATP7B*) gene [1–4]. Excess copper accumulates in tissues throughout the body and, if not treated, can lead to irreversible organ damage and death [2, 4].

Healthcare professionals face numerous challenges in confirming a suspected diagnosis of WD, including variable disease presentations, the rarity of the disease [1, 3], and possibly reduced penetrance [5]. It manifests with heterogenous signs and symptoms that can resemble and overlap with more common conditions and present alone or in combination. The most common manifestations are hepatic and/or neuropsychiatric, but ophthalmological, hematological, renal, cardiac, and musculoskeletal signs and symptoms can also occur [6–12].

Early diagnosis and treatment are critical to avoid progression to advanced or irreversible hepatic, neurological, and/or psychiatric symptoms. Timely diagnosis of WD can affect patient outcomes: In particular, cirrhosis at the time of diagnosis is associated with worse long-term outcomes [13]. However, diagnostic errors are common and likely delay accurate diagnosis and treatment/management plans [8, 12–17]. The time between initial symptoms and diagnosis can exceed 5 years. Patients who present with neurological rather than hepatic symptoms receive a diagnosis significantly later and are at a greater risk of misdiagnosis [16, 18]. In particular, a patient survey indicated that patients with neurological presentation received an incorrect diagnosis in 48% of cases [18]. Initial misdiagnosis of WD includes over 100 different entities of which Parkinson's disease, essential tremor, and dystonia are the most common [19]. This suggests that guidance on the minimal cluster of signs and symptoms of WD is needed to raise suspicion of WD among healthcare professionals.

Synthesizing screening criteria from gastroenterology and neurology settings into one resource could support healthcare professionals in the timely diagnosis of WD, ultimately leading to better outcomes for patients. Three practice guidelines for WD provide criteria for diagnosis and recommendations for management but can be difficult to implement in clinical practice, likely due to the lack of robust published evidence in the literature [20–23]. Expert consensus could complement these guidelines by providing the best recommendations tailored to current US clinical practice. The modified Delphi technique is a robust and rigorous method to gather group opinion and achieve consensus and has been used widely in healthcare research [24, 25]. We conducted a modified Delphi panel to develop consensus on minimal screening criteria for the diagnosis of WD in gastroenterology and neurology settings in the United States that can be implemented by healthcare professionals in clinical practice.

## 2. Methods

### 2.1. Study Design

In this modified Delphi panel study (Figure S1), we developed an initial survey based on input from a targeted literature review and a steering committee (SC) of four clinical experts in WD (two gastroenterologists/hepatologists [R.P.G.-P. and V.M.] and two neurologists [J.M.B. and M.L.]). The SC determined the panelist inclusion criteria, Round 1 survey content, and analysis rules (Table 1). Consensus statements were refined during three rounds of surveys (March–September 2021) with a separate panel of expert clinicians [26]. In each round, panelists rated the extent of their agreement with each statement. The predetermined consensus level was 80%. Additional information is provided in the supporting methods.

### 2.2. Panelist Selection

Purposive sampling was used to recruit 32 panel participants, who were chosen according to their expertise within their specific field [27, 28]. Clinicians who were selected to participate had to meet the following prespecified inclusion criteria: (1) be a practicing gastroenterologist/hepatologist or neurologist in the United States, (2) have seen at least one adult or pediatric patient with WD in the last 2 years, and (3) have at least one publication on WD. Once participants provided written consent, Round 1 interviews were arranged.

### 2.3. Survey Development

**Figure 1:**
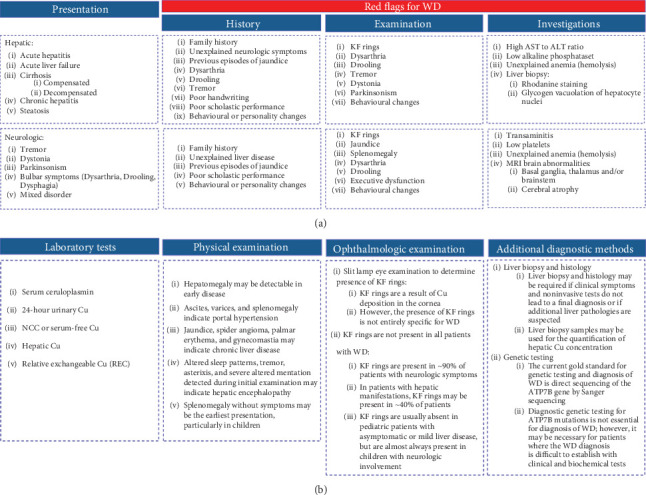
Summary of (a) common hepatic and neurologic presentations of WD and associated red flags that should prompt investigation for WD and (b) tests and examinations for the diagnosis of WD. ALT, alanine transaminase; AST, aspartate transaminase; Cu, copper; KF, Kayser–Fleischer; MRI, magnetic resonance imaging; NCC, non–ceruloplasmin-bound copper; WD, Wilson disease.

A targeted literature review was conducted to generate initial statements (Table S1). The SC assessed the findings with respect to real-world clinical practice and described common hepatic and neurologic presentations of WD and associated red flags that should prompt investigation for WD (Figure 1a). Assuming a patient presented with such red flags, the SC described the tests and/or examinations they would undertake to confirm a diagnosis of WD (Figure 1b). Consensus statements were drafted and reviewed to create the first draft of the survey, which the SC approved.

The survey focused on four domains: the clinical features of WD, the combined clinical findings and signs or symptoms that would raise a suspicion for WD, the minimal diagnostic tests to confirm or establish a diagnosis of WD, and the management of and multidisciplinary approach towards WD (Table S2).

In each round, participants rated the extent of their agreement with each statement. After every round, survey responses for each statement were entered into a Microsoft Excel database. For Round 1, panelists used either quantitative (5-point Likert scale) or qualitative (open-ended) response options that allowed them to provide response rationales. Panelists were also asked to rank the order of tests they would conduct to confirm a diagnosis of WD (from first to last). In Rounds 2 and 3, participants used qualitative (3-point Likert scale or binary) response options per the analysis rules. The diagnostic tests were arranged in six different stages according to the comments and ratings provided by the panel during Round 1. Panelists were asked to indicate their level of agreement with each stage in order of test execution, with the intention of providing a general recommendation rather than substituting the clinical decisions of the treating clinician for specific cases.

### 2.4. First Round

During Round 1, panelists participated in audio-recorded, 1:1 teleconference interviews with researchers to complete a paper-based survey containing 70 statements and 12 open-ended questions. The qualitative comments received from panelists during Round 1 were used to improve survey statements for Round 2.

### 2.5. Second and Third Rounds

Following analysis of Round 1 responses, 56 statements were created from qualitative comments, specific pre-existing statements were split for clarity, and 7 statements that did not achieve the required response threshold were removed. The rank order question and all open-ended questions were removed, leading to the creation of a 119-item survey for Round 2, which the SC approved before it was sent to panelists.

Rounds 2 and 3 were conducted via interactive PDF questionnaires emailed to participants, who were given 14 days to respond. In Rounds 2 and 3, the results of the previous round were shared with panelists, including the panelists' own previous answer and the interquartile range, mode, and mean response that each statement received from all panelists in the previous round. Quantitative data collected during Round 2 were analyzed and used to develop the Round 3 questionnaire, leading to the removal of 10 statements not reaching the required response threshold and the creation of an 84-item survey for Round 3. Following Round 3 data analysis, 69 statements achieved consensus, and 15 statements were removed.

## 3. Results

### 3.1. Survey Participation

Twelve US clinicians accepted the invitation to participate in the modified Delphi panel. Of these, 11 panelists participated in all three rounds (six gastroenterologists/hepatologists and five neurologists).

### 3.2. Consensus Statements

**Figure 2:**
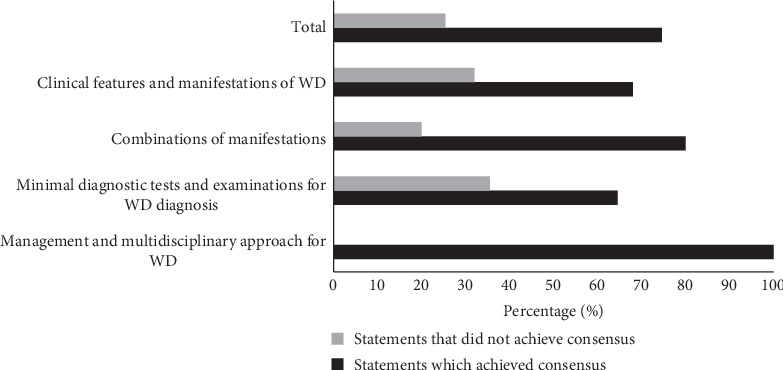
Percentage of statements that did/did not achieve consensus, per domain. WD, Wilson disease.

In total, 94 of 126 (74.60%) statements achieved consensus (Figure 2). The “Management and Multidisciplinary Approach for WD” (100%) and “Combinations of Manifestations” (80%) domains had the highest percentages of consensus.

#### 3.2.1. Clinical Features and Manifestations of WD

The panelists agreed on 17 of 25 (68%) statements relating to general clinical manifestations of WD. There was unanimous agreement that isolated hepatic involvement is more common in childhood and adolescence than adulthood, indicating an age-related phenotypic nature of the disease. Consensus was achieved for the belief that acute severe hemolysis and encephalopathy can be seen among initial manifestations of WD-related acute liver failure (ALF).

Panelists agreed that neuropsychiatric symptoms can be the sole presenting clinical symptom of WD and that clinical symptoms of WD vary significantly, affecting various neurological domains, including tremors, dystonia, seizures, parkinsonism, ataxia, cognitive changes, and behavioral issues. Panelists agreed that young-onset parkinsonism can be a neurological manifestation of WD and that unexplained new onset neuropsychiatric features (e.g., psychosis, severe depression, anxiety, bipolar disorder, ADHD, and autism spectrum disorder) may be manifestations of WD. Agreement was achieved that a change/decline in school/work performance can be a neuropsychiatric manifestation of WD. Three renal manifestations achieved consensus: (1) Renal tubular dysfunction with nephrocalcinosis (manifesting as microscopic hematuria) can occur in WD. (2) Renal tubular dysfunction with nephrocalcinosis (manifesting as microscopic hematuria) is uncommon in pediatric WD. (3) Fanconi syndrome resulting in aminoaciduria can occur as a presentation of WD but is uncommon. Finally, joint manifestations (e.g., osteoarthritis and chondrocalcinosis) may be symptoms of WD and should raise suspicion of WD if presenting in the second and third decades (Table S3).

Statements on clinical features of WD that did not reach consensus were primarily concerning hepatic manifestations (Table S4). Panelists did not reach consensus on whether the neuropsychiatric symptoms of WD usually present later than the hepatic symptoms.

#### 3.2.2. Combinations of Manifestations

**Figure 3:**
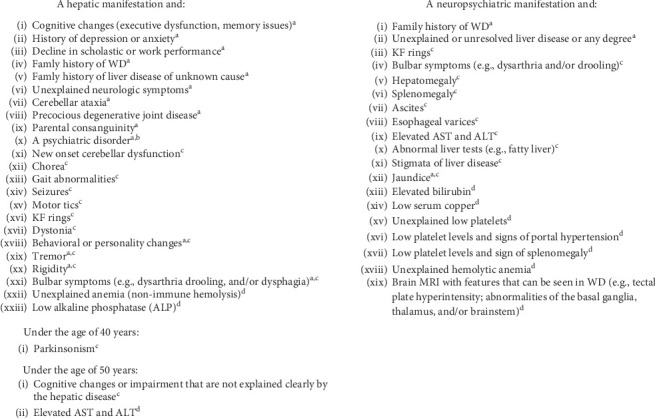
Statements reaching consensus on combinations of clinical manifestations and findings from patient medical and/or family history, physical examinations, or previous evaluations that should prompt investigation for WD. ALT, alanine transaminase; AST, aspartate transaminase; KF, Kayser–Fleischer; MRI, magnetic resonance imaging; WD, Wilson disease. ^a^Patient medical and/or family history. ^b^Psychosis, bipolar disorder, schizoaffective disorder, attention deficit hyperactivity disorder, and autism spectrum disorder. ^c^Condition disclosed by patient's examination. ^d^Result disclosed by patient's previous workup/routine exam/investigation.

Panelists agreed on 52 out of 65 (80%) combinations of hepatic (*n* = 31) or neuropsychiatric (*n* = 21) manifestations and findings from a patient's medical and/or family history, physical examinations, or previous evaluation or routine examination/investigation that should prompt an investigation for WD (Figure 3).

For example, panelists agreed that WD should be investigated in patients with a hepatic manifestation and (1) a medical/family history of unexplained neurological symptoms (e.g., bulbar symptoms, tremor, rigidity, cognitive changes, and cerebellar ataxia) or (2) the presence of neurological signs (e.g., tremor, rigidity, dystonia, bulbar symptoms, gait abnormalities, parkinsonism under the age of 40 years old, chorea, new onset cerebellar dysfunction, motor ticks, and seizures) upon physical examination.

Statements on combined clinical findings and manifestations of WD that did not reach consensus were primarily about hepatic manifestations, though there were a few statements regarding laboratory tests and imaging studies for patients with neuropsychiatric manifestations (Table S4).

#### 3.2.3. Minimal Diagnostic Tests and Examinations for WD Diagnosis

All panelists agreed on 9 out of 17 (52.94%) minimal diagnostic tests and/or examinations to confirm WD diagnosis. These included serum ceruloplasmin and 24-h urine copper testing (values < 10 mg/dL and > 100 *μ*g/24 h, respectively, favor the diagnosis of WD), examination for KF rings (e.g., optical tomography and slit lamp examination), checking for stigmata of liver disease, a comprehensive neurologic exam, assessing for a family history of WD, a brain MRI (if there are neurological findings), a complete blood count, and a hepatic panel (alkaline phosphatase [ALP], aspartate aminotransferase, alanine aminotransferase, and bilirubin).

**Figure 4:**
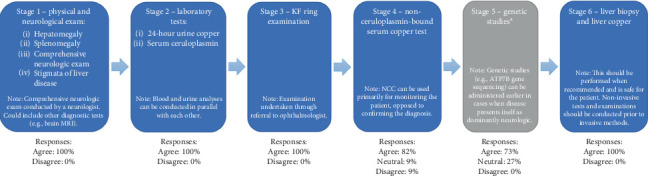
Stages in order of execution for the diagnosis confirmation of WD. KF, Kayser–Fleischer; MRI, magnetic resonance imaging; NCC, non–ceruloplasmin-bound copper; WD, Wilson disease. ^a^Consensus was not achieved on the position of genetic studies in the stages of confirming WD diagnosis.

Panelists agreed on five stages in order of execution to confirm a WD diagnosis (Figure 4). Consensus was reached on the role of genetic studies as a diagnostic test, but not on its position in the order of execution. Consensus was achieved on the role of estimated non–ceruloplasmin-bound copper (NCC) and physical examinations in sequence, but they failed to reach consensus as stand-alone statements.

Panelists agreed upon six statements concerning nuances and practices related to minimal diagnostic tests: (1) Noninvasive tests should be preferred before invasive tests for the diagnosis of WD, (2) the presence of KF rings needs to be confirmed via slit lamp examination but may be observed without special equipment, and (3) the absence of KF rings does not exclude a diagnosis of WD. Other nuances included that (4) screening for WD should be considered in any child/young adult presenting with unexplained new onset neuropsychiatric features, (5) ATP7B mutation analysis is recommended as a clinical diagnostic test to support the diagnosis of WD in a patient suspected to have WD, and (6) with 24-h urine copper tests, lower levels (> 40 *μ*g/24 h) have been recommended especially for asymptomatic siblings but is less specific.

#### 3.2.4. Management and Multidisciplinary Approach for WD

Consensus was reached on all five statements (100%) regarding the management of and multidisciplinary approach to WD. Panelists agreed that patients on D-penicillamine and trientine should be periodically evaluated for proteinuria to detect drug-induced glomerular injury. Panelists also agreed that the disappearance of KF rings often correlates with adequate copper removal (e.g., chelation) but may take years.

Panelists agreed on two methods that specify the need for a multidisciplinary approach to WD diagnosis. When assessing clinical symptoms or signs that are not in keeping with your understanding of WD, it is important to (1) collaborate in a multidisciplinary team and/or refer to another specialty (e.g., ophthalmologist, neurologist, and hepatologist) when available and (2) refer to a gastroenterologist/hepatologist if you are a neurologist and to a neurologist if you are a gastroenterologist/hepatologist.

## 4. Discussion

Using a modified Delphi panel, we obtained expert consensus on minimal signs/symptoms to raise a suspicion of WD and a minimal screening approach for WD diagnosis that can be implemented by practicing US healthcare professionals. Screening should include serum ceruloplasmin and 24-h urine copper measurement. Panelists agreed that diagnosis should be confirmed using physical examination (including neurological and KF ring examination), laboratory tests (including ceruloplasmin), and, in some cases, liver biopsy. Panelists agreed that the absence of KF rings does not exclude WD. Patient age was mentioned several times across topics, highlighting the age-related phenotypic nature of WD. Panelists agreed that there was a wide range of combinations (e.g., behavior/personality changes and psychiatric disorders) rather than just one specific neuropsychiatric presentation.

Although panelists agreed on the use of ATP7B variant testing to support a diagnosis of WD, they did not reach consensus on when it should be performed. There is likely disagreement on the timing of the genetic test (e.g., whether it should be conducted early as a screening tool, or later in difficult-to-confirm cases) or who should interpret the results. Although clinicians' knowledge about disease-causing variants is growing, geneticists can provide additional support, especially for families with multiple family members who need to be screened. The SC notes that ATP7B mutation analysis is especially recommended when a diagnosis of WD remains in doubt. The panelists also agreed with a statement that lower levels (> 40 *μ*g/24 h) for 24-h urine copper tests have been recommended, especially for asymptomatic siblings, but are less specific. The SC acknowledges that this statement is different from the well-established diagnostic criteria for WD (e.g., Leipzig score) and wishes to explain that it was included as a reflection of panelists' opinions on the subject, rather than an attempt to rewrite the criteria.

The Delphi panel offers a unique perspective as it can complement medical society–endorsed guidelines by identifying controversies, discrepancies, and knowledge gaps among practitioners. This study is a valuable intersection between current clinical practice among gastroenterologists/hepatologists and neurologists and the point of view of experts in the field. Several important aspects in diagnosing WD were highlighted, including the use of serum ceruloplasmin and 24-h urine copper to screen for WD. The SC suggests that serum ceruloplasmin and 24-h urine copper testing are sufficient for screening patients when WD is being considered. If results from these screening tests are abnormal or there still is suspicion that the patient has WD, further testing is indicated and should include the other minimal diagnostic tests/examinations that reached consensus (i.e., a comprehensive neurologic exam, examination for KF rings and stigmata of liver disease, complete blood count, hepatic panel, brain MRI, and assessing for family history of WD). Although the panelists recommended NCC testing, calculated NCC can often yield invalid values and cannot be relied upon as a screening tool [2]. Due to the lack of a reliable way of measuring NCC, the 2022 American Association for the Study of Liver Diseases multidisciplinary guidance on the diagnosis and management of WD has de-emphasized the role of calculated NCC [21], and this is reflected in the outcomes of this Delphi panel as individual NCC statements failed to reach consensus. Direct measurement of NCC is currently used only in research settings but may become more widely available in the future. In addition, the SC notes that NCC is not adequate for monitoring alone.

The panelists preferred noninvasive over invasive tests. General efforts in hepatology are leading to attempts to avoid invasive procedures when possible, a shift that is also potentially captured in the European Association for the Study of the Liver and European Society for Paediatric Gastroenterology Hepatology and Nutrition clinical practice guidelines for WD. However, more effort is needed to clarify which noninvasive tests should be regularly used in clinical practice. One interesting statement reaching consensus in this panel was regarding the significance of low ALP levels in prompting an investigation for WD in patients with hepatic manifestations. The potential diagnostic importance of low ALP has been demonstrated only in WD-related ALF WD [21, 29]. These findings suggest a need for more studies on the significance of ALP levels in WD-associated chronic liver disease. There was less consensus among panelists regarding extrahepatic and nonneurologic manifestations of WD. More research on those manifestations is needed to inform clinical practice and future guidelines [21].

The present Delphi panel study is subject to certain limitations. Given the rare nature of the disease, the panel was recruited based on recommendations from the SC and study sponsor, creating a set of expert views which potentially lacked variation in practices and opinions. Due to the small sample size, the Round 1 survey did not undergo any pilot testing. The panelists were asked to respond to each statement regardless of their speciality (e.g., gastroenterologists responding to statements about neurological presentations and vice versa) and were unable to indicate instances when they did not know the answer. No demographic or clinical practice information (e.g., practice setting and number/age of patients with WD seen) was collected; therefore, our knowledge of the sample is restricted. Differences in practice setting (e.g., academia and public or private institutions) may contribute to variability in the panelists' experience and/or access to resources such as multidisciplinary teams.

The results of this modified Delphi panel provide a practical approach towards considering WD as a potential diagnosis, including empirical insight on the minimal signs and symptoms to raise a suspicion of WD and the minimal tests to confirm or rule out a diagnosis of WD. It also provides information on the diagnostic testing habits of healthcare providers, disjointed from guidelines. These results can complement the existing clinical guidance on WD diagnosis with the real-world experience of practicing gastroenterologists/hepatologists and neurologists. The methods for cross-specialty collaboration identified by the Delphi panelists can help providers connect nonspecific comorbid symptoms and accelerate diagnosis. We hope that the results give a wide audience of providers a simple approach to diagnose WD to reduce diagnostic delay and avoid irreversible organ damage and other negative impacts of untreated WD.

## Figures and Tables

**Table 1:** Analysis rules.

Rule 1: Questions that show variable response patterns (≤ 40%) spread across response options in a nonskewed way will be removed
Rule 2: Questions with responses between 41% and 79% will be asked again with three response options: disagree, neither agree nor disagree, and agree
Rule 3: Questions that showed a skewed response pattern, with majority of responses (≥ 80%) spread across five or three options, will be summarized and asked back with a binary response option (agree/disagree)
Rule 4: Binary questions that showed a response pattern of ≥ 80% agreement will be considered consensus
Rule 5: 3-point Likert scale questions in the third round with ≥ 80% of a response option will be considered consensus
Rule 6: 3-point Likert scale questions in Round 2 with responses between 41% and 79% will be reasked on a 3-point Likert scale in Round 3^a^

^a^ ^a^Rule 6 was added during Round 2 analysis to be able to analyze 3-point Likert scale questions.

## Data Availability

The data that support the findings of this study are available from Alexion Pharmaceuticals Inc. Restrictions apply to the availability of these data, which were used under license for this study. Data are available from the authors with the permission of Alexion Pharmaceuticals Inc.

## Supporting Information

Supporting Information Additional supporting information can be found online in the Supporting Information section. The supporting information file includes the following: (1) supporting methods, including study design, panelist selection, preparation, data analysis, and related references. (2) Figure S1. Modified Delphi panel process. (3) Table S1. Search string. (4) Table S2. Domain and subdomains assessed by the survey. (5) Table S3. Statements that reached consensus. (6) Table S4. Statements that did not reach consensus.
