# Supplementary material for: Minimal Criteria to Screen for Wilson Disease: A Delphi Consensus in the United States
Source: Int J Hepatol. 2025 Nov 1;2025:5525442. doi: 10.1155/ijh/5525442 (PMC12596148; doi:10.1155/ijh/5525442)
Supplement: Supporting Information — Additional supporting information can be found online in the Supporting Information section. The supporting information file includes the following: (1) supporting methods, including study design, panelist selection, preparation, data analysis, and related references. (2) Figure S1. Modified Delphi panel process. (3) Table S1. Search string. (4) Table S2. Domain and subdomains assessed by the survey. (5) Table S3. Statements that reached consensus. (6) Table S4. Statements that did not reach consensus. [file 5525442.f1.docx]

# SUPPLEMENTAL MATERIAL

## Supplemental Methods

**Study Design**

The Delphi method is an iterative technique characterized by repeated rounds of anonymous controlled feedback until consensus is achieved.^1^ It allows the systematic collection and aggregation of informed judgements from experts. The Delphi method is a practical way to gain consensus in situations where evidence and standard research pathways are limited.^2^

**Panelist Selection**

Based on estimates suggesting a sample between 5 and 20 individuals is adequate, 32 clinicians from the United States (US) were invited via email to participate as panelists.^3^ Clinicians fulfilling the inclusion criteria were contacted by email invite detailing the participation activities, the reason for their invitation, and the study objective.

**Preparation**

In December 2020 and January 2021, the steering committee (SC) were convened to determine the panelist inclusion criteria and content of the round 1 survey, identify potential panelists, and agree upon the analysis rules, which were later refined and standardized.

To guide steering committee (SC) discussion and generate initial statements, we conducted a targeted literature review of all available guidelines from around the world and key articles on screening criteria for Wilson disease (WD) in the gastroenterology and neurology settings. The literature search was conducted in MEDLINE and EMBASE simultaneously by means of the ProQuest portal (accessed November 17, 2020). The search was limited to English-language practice guidelines and case studies for diagnosis and/or management of WD in the gastroenterology and/or neurology setting. The search strategy combined relevant search terms comprising indexed keywords and terms appearing in the title and abstract of the database records (**eTable 1**).

A total of 260 publications were identified, of which 50 publications were eligible for full-text screening. Top-line data were extracted into a spreadsheet in Microsoft Excel. Data extracted included population, study setting, methods (only relevant for case studies), clinical presentation, diagnostic approach/screening criteria, relevant tables or figures on diagnostic screening pathway, and key recommendations/conclusions.

A total of 10 publications formed the basis of the draft framework development.^4-13^ Findings of the literature were summarized in 2 tables for the SC to discuss.

**Data Analysis**

For quantitative analysis, the SC agreed upon an a priori consensus level of 80% (i.e., requiring ≥80% of panelists to answer the same way). After every round, survey responses for each statement were entered into a Microsoft Excel database. For Likert scale statements, levels of dispersion (IQR), central tendencies (mean, median, mode), and percentage frequencies of responses to each statement were calculated between each round. For the rank order statement in round 1, panelists’ responses were aggregated to form scores for each test which were then ordered from highest to lowest (highest being the tests panelists would conduct first). Tests/examinations which panelists indicated they would execute at the same time were then grouped to form 6 stages.

**References**

1. Hsu C-C, Sandford BA. The Delphi technique: making sense of consensus. *Practical Assess Res Eval*. 2007;12:10. doi:10.7275/pdz9-th90

2. Nasa P, Jain R, Juneja D. Delphi methodology in healthcare research: how to decide its appropriateness. *World J Methodol*. 2021;11(4):116-129. doi:10.5662/wjm.v11.i4.116

3. Rowe G, Wright G. Expert Opinions in Forecasting: The Role of the Delphi Technique. In: Armstrong JS, ed. *Principles of Forecasting: A Handbook for Researchers and Practitioners*. Springer US; 2001:125-144.

4. Poujois A, Woimant F. Challenges in the diagnosis of Wilson disease. *Ann Transl Med*. 2019:13.

5. Palumbo CS, Schilsky ML. Clinical practice guidelines in Wilson disease. *Ann Transl Med*. 2019:11.

6. European Association for the Study of the Liver. EASL Clinical Practice Guidelines: Wilson's disease. *J Hepatol*. 2012;56(3):671-685. doi:10.1016/j.jhep.2011.11.007

7. Roberts EA, Schilsky ML. Diagnosis and treatment of Wilson disease: an update. *Hepatology*. 2008;47(6):2089-2111. doi:10.1002/hep.22261

8. Fernando M, van Mourik I, Wassmer E, Kelly D. Wilson disease in children and adolescents. *Arch Dis Child*. 2020;105(5):499-505. doi:10.1136/archdischild-2018-315705

9. Nagral A, Sarma MS, Matthai J, et al. Wilson's disease: Clinical Practice Guidelines of the Indian National Association for Study of the Liver, the Indian Society of Pediatric Gastroenterology, Hepatology and Nutrition, and the Movement Disorders Society of India. *J Clin Exp Hepatol*. 2019;9(1):74-98. doi:10.1016/j.jceh.2018.08.009

10. Ryan A, Nevitt SJ, Tuohy O, Cook P. Biomarkers for diagnosis of Wilson's disease. *Cochrane Database Syst Rev*. 2019;2019(11). doi:10.1002/14651858.CD012267.pub2

11. Shribman S, Warner TT, Dooley JS. Clinical presentations of Wilson disease. *Ann Transl Med*. 2019:6.

12. Sintusek P, Chongsrisawat V, Poovorawan Y. Wilson's disease in Thai children between 2000 and 2012 at King Chulalongkorn Memorial Hospital. *J Med Assoc Thai*. 2016;99(2):182-187.

13. Socha P, Janczyk W, Dhawan A, et al. Wilson's disease in children: a position paper by the Hepatology Committee of the European Society for Paediatric Gastroenterology, Hepatology and Nutrition. *J Pediatr Gastroenterol Nutr*. 2018;66(2):334-344. doi:10.1097/mpg.0000000000001787

**Figure S1. Modified Delphi panel process**


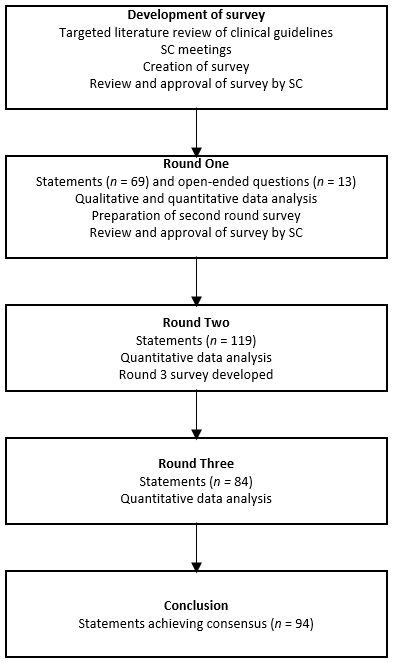


**Abbreviation:** SC, steering committee.

**Table S1. Search strings**

| Topic | Search | Searched for | # hits |
| --- | --- | --- | --- |
| Condition | S1 | TI,AB(Wilson's disease) | 11,401* |
|  | S2 | TI,AB(Wilsons disease) | 17,508* |
|  | S3 | TI,AB(wilson syndrome) | 2208° |
|  | S4 | TI,AB(wilson degeneration) | 794° |
|  | S5 | TI,AB(progressive lenticular degeneration) | 65° |
|  | S6 | TI,AB(hepatocerebral degeneration) | 268° |
|  | S7 | TI,AB(hepatolenticular degeneration) | 913° |
|  | S8 | TI,AB(hepatolenticular syndrome) | 84° |
|  | S9 | TI,AB(morbus wilson) | 303° |
|  | S10 | TI,AB(Westphal-Strumpell Syndrome) | 10° |
|  | S11 | EMB.EXACT("Wilson disease") | 12,144* |
|  | S12 | MESH.EXACT("Hepatolenticular Degeneration") | 5859* |
|  | S13 | S1 OR S2 OR S3 OR S4 OR S5 OR S6 OR S7 OR S8 OR S9 OR S10 OR S11 OR S12 | 23,773* |
| Diagnosis & Management | S14 | TI,AB(criteria) OR TI,AB(symptom*) | 4,514,782* |
|  | S15 | TI,AB(Diagnosis) OR TI,AB(Diagnostic) OR TI,AB(Screening) | 6,321,915* |
|  | S16 | TI,AB(Management) OR TI,AB(Assessment) OR TI,AB(Pathway) | 7,632,034* |
|  | S17 | S14 OR S15 OR S16 | 15,325,355* |
| Setting | S18 | TI,AB(Gastroenterology OR Gastric OR Gastroenterologist OR Hepatology OR Hepatologist) | 703,538* |
|  | S19 | TI,AB(Neurology OR Neurological OR Neurologist) | 665,342* |
|  | S20 | S18 OR S19 | 1,365,152* |
|  | **S21** | **S13 AND S17 AND S20** | **1729°** |
| Animal studies | S22 | MESH.EXACT("Animals") | 6,703,032* |
|  | S23 | EMB.EXACT("animal") | 1,986,153* |
|  | S24 | EMB.EXACT("nonhuman") | 6,409,452* |
|  | S25 | ti,ab,su(rat OR rats OR mouse OR mice OR murine OR rodent OR rodents OR hamster OR hamsters) | 7,810,877* |
|  | S26 | S22 OR S23 OR S24 OR S25 | 16,391,074* |
| Human studies | S27 | EMB.EXACT.EXPLODE("human") | 23,238,702* |
|  | S28 | S26 AND S27 | 2,656,507* |
| Exclusion of pure animal studies | S29 | S26 NOT S28 | 13,734,567* |
| Exclusion of conference abstracts/posters older than last 3 years | S30 | PSTYPE("Conference proceedings") AND pd(<20180101) | 4327° |
|  | S31 | RTYPE("Conference abstract") AND pd(<20180101) | 3,016,990* |
|  | S32 | S29 OR S30 OR S31 | 16,489,381* |
|  | S33 | S21 NOT S32 | 1291° |
| English language | S34 | la.exact("English") | 59,836,765* |
|  | **S35** | **S33 AND S34** | **358°** |
| Treatment patterns/ guidelines | S36 | RTYPE("practice guideline") OR EMB.EXACT.EXPLODE("practice guideline") OR MESH.EXACT("Practice Guidelines as Topic") OR TI,AB("practice guideline") OR TI,AB("practice guidance") OR TI,AB((treatment OR therapy OR clinical OR care OR consensus) NEAR/3 (guideline* OR guidance* OR pathway* OR protocol* OR pattern*)) OR TI,AB(clinical OR treatment OR therapy OR guideline) NEAR/3 TI,AB("consensus") OR EMB.EXACT("clinical pathway") OR MESH.EXACT("Clinical Protocols") OR MESH.EXACT("Critical Pathways") OR MESH.EXACT("Standard of Care") OR TI,AB("disease management") OR TI,AB("standard of care" OR "standards of care") | 1,298,861* |
| Case studies | S37 | TI,AB(case NEAR/1 (stud* OR report OR serie*)) OR EMB.EXACT("Case study") OR RTYPE("Case reports") | 3,665,687* |
|  | S38 | S36 OR S37 | 4,912,780* |
| Total | **S39** | **S38 AND S35** | **260** |

* Duplicates are removed from the search, but included in the result count.° Duplicates are removed from the search and from the result count.

**Table S2. Domains and sub-domains assessed by the survey**

| Domain | Sub-domain |
| --- | --- |
| Clinical features of WD | Hepatic |
|  | Neuropsychiatric |
|  | Renal |
|  | Ocular |
|  | Hematological |
|  | Other system manifestations |
| Combinations of manifestations | A hepatic manifestation and specific medical and/or family history |
|  | A hepatic manifestation and specific findings from a patient examination |
|  | A hepatic manifestation and specific findings from a patient’s previous workup/routine exam/investigation |
|  | A neuropsychiatric manifestation and specific medical and/or family history |
|  | A neuropsychiatric manifestation and specific findings from a patient examination |
|  | A neuropsychiatric manifestation and specific findings a from patient’s previous workup/routine exam/investigation |
| Minimal diagnostic tests and examinations undertaken for WD diagnosis | Minimal diagnostic tests and/ or examinations undertaken for the diagnosis of WD |
|  | Screening and features of diagnostic tests and examinations for the diagnosis of WD* |
| Management and multidisciplinary approach for WD^a^ | No sub-domains |

^a^ Added in round 2.

**Table S3.** **Statements That Reached Consensus (≥80% Agreement)**

**Section 1: Consensus Statements on Clinical Features of WD**

| Statement | Response option | Frequency (%) |
| --- | --- | --- |
| Neurological/neuropsychiatric symptoms can be the sole presenting clinical symptom of WD | Binary scale | Agree: 100  Disagree: 0 |
| The clinical symptomatology of WD varies significantly affecting various neurological domains, including mild tremors, dystonia, seizures, parkinsonism, ataxia, cognitive changes, and behavioral issues | Binary scale | Agree: 100  Disagree: 0 |
| Isolated hepatic involvement is more common in childhood and adolescence than adulthood demonstrating an age phenotypic nature of the disease | Binary scale | Agree: 100  Disagree: 0 |
| Acute-on-chronic liver failure (ACLF), acute hepatitis, asymptomatic elevations of aspartate transaminase (AST) and alanine transaminase (ALT), fatty liver, and rarely hepatobiliary malignancies are also manifestations of hepatic WD | Binary scale | Agree: 100  Disagree: 0 |
| Hepatic manifestations of WD can include chronic hepatitis occurring after acute hepatitis | Three-point Likert scale | Agree: 91  Neutral: 9  Disagree: 0 |
| Young onset parkinsonism can be a neuropsychiatric manifestation of WD | Binary scale | Agree: 100  Disagree: 0 |
| A change/decline in school/work performance can be a neuropsychiatric manifestation of WD | Three-point Likert scale | Agree: 100  Neutral: 0  Disagree: 0 |
| Unexplained, new onset neuropsychiatric features (e.g. psychosis, severe depression, anxiety, bipolar disorder, ADHD, Autism Spectrum Disorder) may be manifestations of WD | Three-point Likert scale | Agree: 100  Neutral: 0  Disagree: 0 |
| In adult patients, Kayser–Fleischer (KF) rings are usually bilateral and present in many cases of neurological WD and in around half of hepatic WD | Binary scale | Agree: 100  Disagree: 0 |
| Sunflower cataract is rare, even in neurological WD, and requires slit lap evaluation | Binary scale | Agree: 91  Disagree: 9 |
| Renal tubular dysfunction with nephrocalcinosis (manifesting as microscopic hematuria) can occur in WD | Three-point Likert scale | Agree: 82  Neutral: 18  Disagree: 0 |

**Section 2: Combined Clinical Findings and Manifestations**

| **Statement** | **Response option** | **Frequency (%)** |
| --- | --- | --- |
| A patient with a hepatic manifestation and a decline in scholastic or work performance should prompt investigation for WD | Binary scale | Agree: 100  Disagree: 0 |
| A patient with a hepatic manifestation and behavioral or personality changes should prompt investigation for WD | Binary scale | Agree: 100  Disagree: 0 |
| A patient with a hepatic manifestation and cognitive changes (executive dysfunction, memory issues) should prompt investigation for WD | Binary scale | Agree: 91  Disagree: 9 |
| A patient with a hepatic manifestation and a history of depression or anxiety should prompt investigation for WD | Binary scale | Agree: 91  Disagree: 9 |
| A patient with a hepatic manifestation and a psychiatric disorder (e.g. psychosis, bipolar disorder, schizoaffective disorder, attention deficit hyperactivity disorder, and autism spectrum disorder) should prompt investigation for WD | Binary scale | Agree: 100  Disagree: 0 |
| A patient with a hepatic manifestation and precocious degenerative joint disease should prompt investigation for WD | Three-point Likert scale | Agree: 82  Neutral: 18  Disagree: 0 |
| A patient with a hepatic manifestation and a family history of liver disease of unknown cause should prompt investigation for WD | Binary scale | Agree: 100  Disagree: 0 |
| A patient with a hepatic manifestation and parental consanguinity should prompt investigation for WD | Three-point Likert scale | Agree: 91  Neutral: 0  Disagree: 9 |
| A patient with a hepatic manifestation and cerebellar ataxia should prompt investigation for WD | Binary scale | Agree: 100  Disagree: 0 |
| A patient with a hepatic manifestation and KF rings should prompt investigation for WD | Binary scale | Agree: 100  Disagree: 0 |
| A patient with a hepatic manifestation and bulbar symptoms including dysarthria and/ or drooling should prompt investigation for WD | Binary scale | Agree: 100  Disagree: 0 |
| A patient with a hepatic manifestation and tremor should prompt investigation for WD | Binary scale | Agree: 100  Disagree: 0 |
| A patient with a hepatic manifestation and rigidity should prompt investigation for WD | Binary scale | Agree: 100  Disagree: 0 |
| A patient with a hepatic manifestation and dystonia should prompt investigation for WD | Binary scale | Agree: 100  Disagree: 0 |
| A patient with a hepatic manifestation and parkinsonism, under the age of 40 years old, should prompt investigation for WD | Binary scale | Agree: 100  Disagree: 0 |
| A patient with a hepatic manifestation and new onset cerebellar dysfunction should prompt investigation for WD | Binary scale | Agree: 82  Disagree: 18 |
| A patient with a hepatic manifestation and chorea should prompt investigation for WD | Binary scale | Agree: 91  Disagree: 9 |
| A patient with a hepatic manifestation and gait abnormalities should prompt investigation for WD | Three-point Likert scale | Agree: 91  Neutral: 9  Disagree: 0 |
| A patient with a hepatic manifestation and behavioral or personality changes should prompt investigation for WD | Binary scale | Agree: 100  Disagree: 0 |
| A patient with a hepatic manifestation and cognitive changes (executive dysfunction, memory issues) that are not explained clearly by the hepatic disease, under the age of 50 years old should prompt investigation for WD | Binary scale | Agree: 100  Disagree: 0 |
| A patient with a hepatic manifestation and cognitive impairment that is not explained clearly by the hepatic disease, under the age of 50 years old should prompt investigation for WD | Binary scale | Agree: 100  Disagree: 0 |
| A patient with a hepatic manifestation and seizures should prompt investigation for WD | Binary scale | Agree: 82  Disagree: 18 |
| A patient with a hepatic manifestation and elevated AST and ALT, under the age of 50 years old, should prompt investigation for WD | Binary scale | Agree: 91  Disagree: 9 |
| A patient with a hepatic manifestation and low alkaline phosphatase (ALP) should prompt investigation for WD | Three-point Likert scale | Agree: 82  Neutral: 18  Disagree: 0 |
| A patient with a hepatic manifestation and unexplained anemia (non-immune hemolysis) should prompt investigation for WD | Binary scale | Agree: 100  Disagree: 0 |
| A patient with a neuropsychiatric manifestation and family history of WD should prompt investigation for WD | Binary scale | Agree: 100  Disagree: 0 |
| A patient with a neuropsychiatric manifestation and unexplained or unresolved liver disease of any degree should prompt investigation for WD | Binary scale | Agree: 100  Disagree: 0 |
| A patient with a neuropsychiatric manifestation and jaundice should prompt investigation for WD | Binary scale | Agree: 100  Disagree: 0 |
| A patient with a neuropsychiatric manifestation and KF rings should prompt investigation for WD | Binary scale | Agree: 100  Disagree: 0 |
| A patient with a neuropsychiatric manifestation and jaundice should prompt investigation for WD | Binary scale | Agree: 100  Disagree: 0 |
| A patient with a neuropsychiatric manifestation and stigmata of liver disease should prompt investigation for WD | Binary scale | Agree: 100  Disagree: 0 |
| A patient with a neuropsychiatric manifestation and hepatomegaly should prompt investigation for WD | Binary scale | Agree: 100  Disagree: 0 |
| A patient with a neuropsychiatric manifestation and ascites should prompt investigation for WD | Binary scale | Agree: 100  Disagree: 0 |
| A patient with a neuropsychiatric manifestation and elevated AST and ALT should prompt investigation for WD | Binary scale | Agree: 100  Disagree: 0 |
| A patient with a neuropsychiatric manifestation and splenomegaly should prompt investigation for WD | Three-point Likert scale | Agree: 100  Neutral: 0  Disagree: 0 |
| A patient with a neuropsychiatric manifestation and bulbar symptoms including dysarthria and/or drooling should prompt investigation for WD | Binary scale | Agree: 100  Disagree: 0 |
| A patient with a neuropsychiatric manifestation and esophageal varices should prompt investigation for WD | Binary scale | Agree: 100  Disagree: 0 |
| A patient with a neuropsychiatric manifestation and abnormal liver tests (e.g fatty liver) should prompt investigation for WD | Binary scale | Agree: 100  Disagree: 0 |
| A patient with a neuropsychiatric manifestation and low serum copper should prompt investigation for WD | Binary scale | Agree: 100  Disagree: 0 |
| A patient with a neuropsychiatric manifestation and unexplained low platelets should prompt investigation for WD | Binary scale | Agree: 91  Disagree: 9 |
| A patient with a neuropsychiatric manifestation and elevated bilirubin should prompt investigation for WD | Three-point Likert scale | Agree: 100  Neutral: 0  Disagree: 0 |
| A patient with a neuropsychiatric manifestation and low platelet levels and signs of portal hypertension should prompt investigation for WD | Binary scale | Agree: 100  Disagree: 0 |
| A patient with a neuropsychiatric manifestation and low platelet levels and sign of splenomegaly should prompt investigation for WD | Binary scale | Agree: 100  Disagree: 0 |
| A patient with a neuropsychiatric manifestation and unexplained hemolytic anemia should prompt investigation for WD | Binary scale | Agree: 100  Disagree: 0 |
| A patient with a neuropsychiatric manifestation and MRI of brain with features that can be seen in WD, such as tectal plate hyperintensity, should prompt investigation for WD | Binary scale | Agree: 100  Disagree: 0 |
| A patient with a neuropsychiatric manifestation and MRI brain abnormalities that can be seen in WD of the basal ganglia, thalamus and/or brainstem should prompt investigation for WD | Binary scale | Agree: 100  Disagree: 0 |

**Section 3: Minimal Diagnostic Tests and/or Examinations for WD Diagnosis**

| **Statement** | **Response option** | **Frequency (%)** |
| --- | --- | --- |
| A minimal diagnostic test and/or examination to confirm the diagnosis of WD is the serum ceruloplasmin test (value of <10 mg/dl favors the diagnosis of WD) | Binary scale | Agree: 100  Disagree: 0 |
| A minimal diagnostic test and/or examination to confirm the diagnosis of WD is the twenty-four hour urine copper test (value of >100 μg/24hr favors the diagnosis of WD) | Binary scale | Agree: 100  Disagree: 0 |
| A minimal diagnostic test and/or examination to confirm the diagnosis of WD is examination for KF rings (e.g. through optical tomography, slit lamp examination) | Binary scale | Agree: 100  Disagree: 0 |
| A minimal diagnostic test and/or examination to confirm the diagnosis of WD is checking for stigmata of liver disease | Binary scale | Agree: 100  Disagree: 0 |
| A minimal diagnostic test and/or examination to confirm the diagnosis of WD is a comprehensive neurologic exam | Binary scale | Agree: 100  Disagree: 0 |
| A minimal diagnostic test and/or examination to confirm the diagnosis of WD is assessing for a family history of WD | Three-point Likert scale | Agree: 91  Neutral: 9  Disagree: 0 |
| A minimal diagnostic test and/or examination to confirm the diagnosis of WD is an MRI of the brain (if there are neurological findings) | Three-point Likert scale | Agree: 91  Neutral: 0  Disagree: 9 |
| A minimal diagnostic test and/or examination to confirm the diagnosis of WD is a complete blood count (CBC) | Three-point Likert scale | Agree: 91  Neutral: 9  Disagree: 0 |
| A minimal diagnostic test and/or examination to confirm the diagnosis of WD is a hepatic panel (ALP,AST ALT, bilirubin) | Binary scale | Agree: 100  Disagree: 0 |

**Section 4: Screening and Features of Diagnostic Tests and Examinations for WD Diagnosis**

| Statement | Response option | Frequency (%) |
| --- | --- | --- |
| With Twenty-four hour urine copper tests, lower levels (>40 μg/24 hours) have been recommended especially for asymptomatic siblings but is less specific | Three-point Likert scale | Agree: 82  Neutral: 18  Disagree: 0 |
| ATP7B mutation analysis is recommended as a clinical diagnostic test to support the diagnosis of WD in a patient suspected to have WD | Three-point Likert scale | Agree: 91  Neutral: 9  Disagree: 0 |
| Non-invasive tests should be preferred before invasive tests for the diagnosis of WD | Binary scale | Agree: 100  Disagree: 0 |
| The absence of KF rings does not exclude a diagnosis of Wilsons disease | Binary scale | Agree: 100  Disagree: 0 |
| Screening for WD should be considered in any child/young adult presenting with unexplained new onset neuropsychiatric features | Binary scale | Agree: 100  Disagree: 0 |
| The presence of KF rings need to be confirmed via slit lamp examination but may be observed without special equipment | Binary scale | Agree: 100  Disagree: 0 |
| Please indicate your level of agreement for each stage in the order of execution for the diagnosis confirmation of WD. We have arranged the tests in 6 different stages accordingly with the comments and ratings provided by the panel during the first round. Take into consideration that the aim is to provide a general recommendation and won’t substitute the clinical decisions of the treating clinician for specific cases. | | |
| Stage 1- Physical and Neurological Exam:  • Hepatomegaly  • Splenomegaly  • Comprehensive neurologic exam  • Stigmata of liver disease  Notes: Comprehensive neurologic exam conducted by a neurologist. The comprehensive neurologic exam could include other diagnostic tests (e.g., an MRI of the brain) | Binary scale | Agree: 100  Disagree: 0 |
| Stage 2- Laboratory Tests:  • 24-hour urine copper  • Serum Ceruloplasmin  Notes: Blood and urine analyses can be conducted in parallel with each other | Binary scale | Agree: 100  Disagree: 0 |
| Stage 3- KF ring examination  Notes: KF ring examination undertaken through referral to ophthalmologist | Binary scale | Agree: 100  Disagree: 0 |
| Stage 4- Non-ceruloplasmin-bound serum copper (NCC) test  Notes: NCC can be used primarily for monitoring the patient, opposed to confirming the diagnosis | Three-point Likert scale | Agree: 82  Neutral: 9  Disagree: 9 |
| Stage 6- Liver biopsy and liver copper  Notes: This should be performed when recommended and is safe for the patient. Non-invasive tests and examinations should be conducted prior to invasive methods. | Binary scale | Agree: 100  Disagree: 0 |

**Table S4.** **Statements That Did Not Reach Consensus (<80% Agreement)**

| **Statement** | **Frequency, %^a^** | | | | |
| --- | --- | --- | --- | --- | --- |
| **Section 1: Consensus Statements on Clinical Features of WD** | | | | | |
|  | **Completely Agree** | **Agree** | **Neutral** | **Not Agree** | **Completely Disagree** |
| Cirrhosis and portal hypertension is a common presentation in hepatic WD | 25 | 33 | 25 | 17 | 0 |
| Concomitant hemolysis is invariably seen in patients with WD presenting with ALF |  |  |  |  |  |
|  | 33 | 33 | 0 | 33 | 0 |
| The neuropsychiatric symptoms of WD usually present later than the hepatic symptoms |  |  |  |  |  |
|  | 17 | 33 | 33 | 17 | 0 |
| Asymptomatic arrhythmias are common, and therefore, a cardiac evaluation should be routinely performed in all adult patients |  |  |  |  |  |
|  | 17 | 33 | 33 | 17 | 0 |
| Features of progressive autoimmune hepatitis, or autoimmune hepatitis unresponsive to treatment, are hepatic manifestations of WD |  |  |  |  |  |
|  | 18 | 27 | 45 | 9 | 0 |
| Incidental fatty liver changes are among the initial presentations of WD |  |  |  |  |  |
|  | 27 | 36 | 36 | 0 | 0 |
| Due to increased excretion of uric acid, hyperuricemia can occur in an untreated patient with WD |  |  |  |  |  |
|  | 36 | 27 | 36 | 0 | 0 |
| Asymptomatic liver test abnormalities are among the most common presentations of WD | **Agree** | | **Neutral** | **Disagree** | |
|  | 73 | | 27 | 0 | |
| **Section 2: Combined Clinical Findings and Manifestations** | | | | | |
| **Medical and/or family history** |  |  |  |  |  |
| A patient with a hepatic manifestation and _____ should prompt investigation for WD |  |  |  |  |  |
| Kidney stones | **Agree** | | **Neutral** | **Disagree** | |
|  | 73 | | 18 | 9 | |
| Blood in their urine (hematuria) aged between 5-35 years old |  |  |  |  |  |
|  | 73 | | 18 | 9 | |
| **Patient examination** |  |  |  |  |  |
| A patient with a hepatic manifestation and _____ should prompt investigation for WD |  |  |  |  |  |
| New onset, pathological hyperreflexia | **Agree** | | **Neutral** | **Disagree** | |
|  | 73 | | 18 | 9 | |
| Postural orthostatic tachycardia syndrome (POTS) |  |  |  |  |  |
|  | 27 | | 55 | 18 | |
| A patient with a neuropsychiatric manifestation and _____ should prompt investigation for WD |  |  |  |  |  |
| Hyperpigmentation of the skin | **Completely Agree** | **Agree** | **Neutral** | **Not Agree** | **Completely Disagree** |
|  | 36 | 36 | 18 | 0 | 9 |
| **Previous evaluation or routine examination/investigation** |  |  |  |  |  |
| A patient with a hepatic manifestation and _____ should prompt investigation for WD |  |  |  |  |  |
| Low serum uric acid | **Completely Agree** | **Agree** | **Neutral** | **Not Agree** | **Completely Disagree** |
|  | 36 | 27 | 36 | 0 | 0 |
| Proteinuria |  |  |  |  |  |
|  | 27 | 27 | 36 | 9 | 0 |
| High bilirubin to ALP ratio | **Agree** | | **Neutral** | **Disagree** | |
|  | 64 | | 36 | 0 | |
| Hematuria |  |  |  |  |  |
|  | 45 | | 45 | 9 | |
| A patient with a neuropsychiatric manifestation and ____ should prompt investigation for WD |  |  |  |  |  |
| Low white cell count | **Completely Agree** | **Agree** | **Neutral** | **Not Agree** | **Completely Disagree** |
|  | 33 | 33 | 33 | 0 | 0 |
| Low serum albumin |  |  |  |  |  |
|  | 36 | 36 | 27 | 0 | 0 |
| Cerebral atrophy identified on an MRI of the brain | **Agree** | | **Neutral** | **Disagree** | |
|  | 45 | | 45 | 9 | |
| MRI brain abnormalities of mineralization |  |  |  |  |  |
|  | 73 | | 18 | 9 | |
| **Section 3: Minimal Diagnostic Tests and/or Examinations for WD Diagnosis** | | | | | |
| Non-ceruloplasmin-bound copper (NCC) | **Completely Agree** | **Agree** | **Neutral** | **Not Agree** | **Completely Disagree** |
| • Can be calculated by subtracting ceruloplasmin-bound Cu from total serum Cu | 33 | 33 | 25 | 8 | 0 |
| • In most untreated patients with WD, it is >200 μg/L |  |  |  |  |  |
| Splenomegaly without symptoms may be the earliest presentation, particularly in children |  |  |  |  |  |
|  | 33 | 25 | 33 | 0 | 8 |
| A minimal diagnostic test and/or examination to confirm the diagnosis of WD is: |  |  |  |  |  |
| A physical examination assessing for shrunken and small liver (in patients suspected of having advanced WD) | **Completely Agree** | **Agree** | **Neutral** | **Not Agree** | **Completely Disagree** |
|  | 36 | 18 | 18 | 18 | 9 |
| Genetic studies (e.g., ATP7B gene sequencing) | **Agree** | | **Neutral** | **Disagree** | |
|  | 64 | | 18 | 18 | |
| A physical examination checking for hepatomegaly |  |  |  |  |  |
|  | 55 | | 45 | 0 | |
| Liver biopsy and liver copper (concentration >250 μg/g dry, favors the diagnosis of WD) |  |  |  |  |  |
|  | 36 | | 45 | 18 | |
| A physical examination for clubbed fingers |  |  |  |  |  |
|  | 27 | | 64 | 9 | |
| Liver ultrasound |  |  |  |  |  |
|  | 36 | | 64 | 0 | |
| **Section 4: Screening and Features of Diagnostic Tests and Examinations for WD Diagnosis** | | | | | |
| The d-penicillamine challenge test should not be used in the diagnosis of WD in adult patients | **Completely Agree** | **Agree** | **Neutral** | **Not Agree** | **Completely Disagree** |
|  | 36 | 27 | 9 | 18 | 9 |
| If serum ceruloplasmin is low (<10 mg/dl) the treating physician should favor the diagnosis of WD without the need of other tests for diagnosis confirmation |  |  |  |  |  |
|  | 0 | 18 | 18 | 27 | 36 |
| Please indicate your level of agreement for each stage in the order of execution for the diagnosis confirmation of WD. We have arranged the tests in 6 different stages accordingly with the comments and ratings provided by the panel during the first round. Take into consideration that the aim is to provide a general recommendation and won’t substitute the clinical decisions of the treating clinician for specific cases. |  |  |  |  |  |
| Stage 5- Genetic studies | **Agree** | | **Neutral** | **Disagree** | |
| Notes: Genetic studies (e.g., ATP7B gene sequencing) can be administer earlier in cases when disease presents itself as dominantly neurologic. | 73 | | 27 | 0 | |

**Abbreviations**: ALF, acute liver failure; ALP, alkaline phosphatase; Cu, copper; MRI, magnetic resonance imaging; WD, Wilson disease.

^a^The 5-point Likert scale options were 1=Completely disagree, 2=Not agree, 3=Neutral, 4=Agree, 5=Completely agree. The 3-point Likert scale options were 1=Disagree, 2=Neutral, 3=Agree.
